# Moderate alcohol intake and heart rate variability adaptations to high-intensity interval training in healthy young adults: the BEER-HIIT study

**DOI:** 10.3389/fcvm.2026.1920023

**Published:** 2026-08-25

**Authors:** Ginés Navarro-Lomas, Alejandro De-la-O, Manuel Dote-Montero, Manuel J. Castillo, Francisco J. Amaro-Gahete, Abel Plaza-Florido

**Affiliations:** 1 Department of Physiology, Faculty of Medicine, Sport and Health University Research Institute (iMUDS), University of Granada, Granada, Spain; 2 Obesity and Diabetes Clinical Research Section, Phoenix Epidemiology and Clinical Research Branch, National Institute of Diabetes and Digestive and Kidney Diseases, National Institutes of Health, Phoenix, AZ, United States; 3 Instituto de Investigación Biosanitaria, Ibs.Granada, Granada, Spain; 4 Centro de Investigación Biomédica en Red Fisiopatología de La Obesidad y Nutrición (CIBERobn), Instituto de Salud Carlos III, Madrid, Spain; 5 Research Center for Exercise Medicine and Sleep/Pediatric Exercise and Genomics Research Center, Department of Pediatrics, School of Medicine, University of California Irvine, Irvine, CA, United States; 6 Department of Physical Education and Sports, Faculty of Sport Sciences, Sport and Health University Research Institute (iMUDS), University of Granada, Granada, Spain

**Keywords:** alcohol, cardiac autonomic function, exercise, sport, vagal modulation

## Abstract

**Background:**

It is unknown whether daily alcohol consumption influences training effects on heart rate variability (HRV). This study investigated: (i) the effects of a 10-week high-intensity interval training (HIIT) on HRV in healthy young adults; and (ii) the effects of daily alcohol consumption on HRV responses to exercise.

**Methods:**

71 healthy young adults (18–40 years old; 52.1% women) participated in the BEER-HIIT study. We conducted a 10-week (2 days/week) controlled trial (ClinicalTrials.gov ID: NCT03660579). Participants were allocated to 5 groups: a non-training group (N-T) and four HIIT groups. Participants in the training groups chose whether to consume alcohol. Those choosing alcohol were randomly allocated to receive beer (5.4%; T-Beer) or an equivalent amount of alcohol (vodka; T-Ethanol) in sparkling water. Those choosing no alcohol were randomly allocated to receive alcohol-free beer (0.0%; T-Zero) or sparkling water (T-Water). Training comprised eight weight-bearing exercises performed in circuit form. HRV was measured before and after the intervention.

**Results:**

No statistically significant between-group differences in HRV outcomes were detected after the intervention (all *p* > 0.05).

**Conclusions:**

Our findings indicate that: (i) no statistically significant differences in HRV responses were detected following a 10-week HIIT program using weight-bearing exercises performed twice per week; and (ii) no statistically significant differences in HRV responses were observed among the assigned beverage intervention groups. These findings should be interpreted cautiously because HRV was a secondary outcome and the study was not specifically powered to detect differences in these parameters.

## 1 Introduction

Heart rate variability (HRV) analyses the differences between successive R-R intervals (1) and is considered a non-invasive biomarker to assess the modulation of the autonomic nervous system on heart function (2). HRV is a psycho-physiological phenomenon affected by several factors and especially by lifestyle (2, 3). Higher vagal-related HRV parameters under resting conditions have been associated with more favorable cardiac autonomic regulation and a lower risk of cardiovascular events (2, 4, 5).

Physical exercise is an integral component of a healthy lifestyle (6) and has been shown to improve cardiac autonomic function by increasing vagal-related HRV parameters (7, 8). The World Health Organization (WHO) recommends that adults undertake 150–300 min of moderate-intensity aerobic physical activity, 75–150 min of vigorous-intensity aerobic physical activity, or an equivalent combination of both, together with muscle-strengthening activities involving the major muscle groups on two or more days per week (9). Nevertheless, many adults do not meet these recommendations, with lack of time frequently reported as an important barrier to regular physical activity (10).

In this context, more time-efficient exercise approaches, such as high-intensity interval training (HIIT), have emerged as alternatives to traditional continuous exercise programs (11, 12). HIIT is characterized by repeated short bouts of vigorous exercise interspersed with periods of passive recovery or low-intensity activity (13, 14). Several HIIT protocols have been shown to improve health-related outcomes, including vagal-related HRV parameters in healthy adults, as reported in a systematic review (15). However, HIIT encompasses a broad range of training methodologies, and the resulting physiological adaptations depend on the specific characteristics of the exercise stimulus, including modality, intensity, interval duration, recovery periods, and number of repetitions (14, 16). Previous research has shown that a whole-body, circuit-based HIIT program involving weight-bearing exercises can improve time-domain HRV parameters in insufficiently active young adults (17). However, it remains unclear whether regular beer or alcohol consumption modifies the HRV adaptations induced by this type of HIIT.

Beer consumption after exercise is a common social practice but may be detrimental because of its alcohol content (18, 19). The physiological effects of alcohol depend on the amount consumed, the frequency of consumption, and the drinking pattern (20). High alcohol intake after an acute bout of exercise has also been associated with unfavorable physiological responses, including impaired muscle function, elevated blood pressure, sleep disruption, and alterations in several biochemical processes (19, 21–23). Although some studies using lower or operationally defined moderate amounts of beer or alcohol have not detected detrimental effects on selected hydration, body-composition, or physical-fitness outcomes (18, 24, 25), these findings should not be interpreted as evidence that alcohol consumption is risk-free, beneficial, or recommended.

Chronic high alcohol intake has been associated with reductions in vagal-related HRV parameters (26). Acute alcohol exposure may also alter HRV through changes in autonomic modulation of sinoatrial discharge and sinoatrial responsiveness to released neurotransmitters (27). By contrast, an observational study in young adults reported higher vagal-related HRV parameters among participants with moderate habitual alcohol consumption than among non-habitual drinkers (28). The authors suggested that this association might partly reflect lower inflammatory activity. However, this cross-sectional association does not demonstrate that alcohol consumption has a beneficial or causal effect on cardiac autonomic regulation. It therefore remains uncertain whether regular alcohol consumption modifies the HRV adaptations elicited by an exercise-training intervention.

Accordingly, the present study aimed to: (i) investigate the effects of a circuit-based HIIT program involving weight-bearing exercises on HRV parameters in healthy young adults; and (ii) determine whether regular consumption of the assigned alcohol-containing beverages influenced HRV responses during the training intervention.

## 2 Material and methods

### 2.1 Human subjects ethical approval statement

The study was conducted in accordance with the latest revision of the Helsinki Declaration, was approved by the Ethics Committee on Human Research at the University of Granada (CEI-Granada; 321-CEIH-2017). Before the study, all participants completed a medical exam and signed an informed consent form.

A total of 71 healthy young adults (18–40 years old; 52.1% women) from the BEER-HIIT trial (ClinicalTrials.gov ID: NCT03660579) (29) were included in this ancillary analysis. The participants were recruited from the province of Granada (Spain), using social networks, local media, and posters. Eligible participants were healthy adults aged 18–40 years, had a body mass index between 18.5 and 30 kg/m^2^, had maintained a stable body mass during the previous 5 months, defined as a change of <3 kg, and were not participating in a structured exercise or weight-loss program. Exclusion criteria were cardiovascular, metabolic, or other clinically relevant disease; an abnormal preparticipation electrocardiogram; pregnancy or lactation; use of medication for chronic conditions; pain, recent injury, or any other condition preventing strenuous exercise; a personal or family history of alcohol-related problems; or unwillingness to comply with the study procedures and allocation process. Before enrolment, all candidates underwent a comprehensive medical examination conducted by a physician. This assessment included a clinical interview and medical history specifically addressing current and previous alcohol consumption, personal or family history of alcohol-related problems, clinical indicators suggestive of harmful alcohol use or dependence, and any medical condition or medication contraindicating alcohol exposure. Only candidates for whom the physician identified no evidence or clinical suspicion of alcohol misuse, alcohol dependence, or another alcohol-related contraindication were eligible. A separate validated alcohol-screening questionnaire, such as the Alcohol Use Disorders Identification Test or CAGE questionnaire, was not administered.

### 2.2 Experimental design

All baseline and post-intervention assessments were conducted between February and May 2018 in the same facilities at the Instituto Mixto Universitario Deporte y Salud (iMUDS), University of Granada. After completing the baseline measurements, participants chose whether they preferred to be included in a non-training (N-T), or one of the four training (T) groups. Those going for training then chose whether they preferred to be included in a group ingesting an ethanol-containing beverage (5.4% alcohol content) or an alcohol-free beverage group. Participants who selected an alcohol-containing beverage were randomly allocated to either the T-Beer group or the T-Ethanol group. Participants who selected an alcohol-free beverage were randomly allocated to either the T-Zero group or the T-Water group. Each group was composed of 8 men and 8 women. This type of non-random (based on individual preference) and random allocation of the participants was conducted following ethical considerations and advice made by the ethical committee (321-CEIH-2017). The participants’ randomization assignment was blinded to the assessment staff.

Dietary habits, habitual physical activity, and usual beverage consumption were assessed using the Mediterranean Diet Adherence Screener (MEDAS) (30), the International Physical Activity Questionnaire (IPAQ) (31), and the Beverage Intake Questionnaire (BEVQ) (32), respectively. All participants were instructed to maintain their usual physical activity levels and not to initiate any additional structured exercise program outside the study intervention. These lifestyle variables were assessed but were not continuously or objectively monitored throughout the 10-week intervention.

#### 2.2.1 Training protocol

A 10-week controlled HIIT intervention was conducted, with training sessions performed twice per week in the late afternoon or early evening from Monday to Friday. At least 48 h of recovery separated consecutive training sessions. Participants assigned to the four training groups were required to attend at least 80% of the prescribed training sessions to be eligible for the per-protocol analyses. Following previously described protocols (29), the weekly training volume ranged from 40 to 65 min. Habitual physical activity was assessed using the International Physical Activity Questionnaire (IPAQ) (31). Participants assigned to the non-training group were instructed to maintain their usual physical activity levels and not to engage in a structured exercise program during the intervention. Training intensity was prescribed based on current evidence regarding HIIT (16). In all cases, exercise intensity was set at >8 on the 0–10 rating of perceived exertion scale (33).

The HIIT intervention was divided into three phases: Phase I, a 4-week familiarization phase; Phase II, lasting 4 weeks; and Phase III, lasting 2 weeks. During Phase I, each session consisted of 2 sets of 16 exercises, with 15–20 s of work at an RPE of 8–9 on a 0–10 scale, intersected with 15–20 s of passive recovery between consecutive exercises within each set. Phase II lasted 4 weeks and consisted of 2 sets of 16 exercises, with 15–30 s of work at an RPE of 10/10 intersected with 15–30 s of passive recovery between consecutive exercises within each set. Finally, phase III lasted 2 weeks, with 3 sets of 16 exercises, with 15–20 s of work at an RPE of 10/10 intersected with 15–20 s of passive recovery between consecutive exercises within each set. Participants performed a 5*-*minute active recovery period at an RPE of 6/10 between sets in each of the three intervention phases.

Training sessions started with a dynamic standardized warm-up composed of several muscle activations exercises: child's pose breathing, pelvis bridge, cat camel, upper back rotation, front and side planks, arms Ts, arms Ys, toe walks, high knee walks, walking lunges, side lunges, monster walk, sumo walk and anti-rotational stability press. Participants performed eight weight-bearing exercises in a circuit form twice per set (i.e., frontal plank, high knees up, TRX horizontal row, battle rope, squat, deadlift, push up, and burpees). Exercise intensity was monitored using a 0–10 rating of perceived exertion (RPE) scale, where 0 represented no exertion and 10 represented maximal exertion. During Phase I, the prescribed exercise intensity was RPE 8–9/10. During Phases II and III, exercise intensity was prescribed at RPE 10/10. Active recovery between sets was performed at RPE 6/10. Training sessions ended with a cooling-down protocol (i.e., active global stretching), including anterior and posterior chain exercises (i.e., pigeon pose, lying twist, figure four stretch, lunging hip flexor stretch, biceps stretch, and trapezius neck stretch).

#### 2.2.2 Beverage intake

The sex-specific beverage volumes were prespecified to provide an ethanol exposure equivalent to approximately two standard drinks per day in men and one standard drink per day in women, in accordance with the operational definition of moderate alcohol intake used when the study protocol was designed (34). One standard drink was defined as approximately 14 g of pure ethanol. Considering the 5.4% alcohol-by-volume content of the regular beer and an ethanol density of approximately 0.789 g/mL, the prescribed volumes of 660 mL/day for men and 330 mL/day for women provided approximately 28 g and 14 g of ethanol, respectively. These doses were selected exclusively to standardize the experimental exposure and should not be interpreted as indicating that alcohol consumption is risk-free or recommended.

Beverages were ingested daily from Monday to Friday. The total fluid volume provided was the same across intervention groups and was sex-specific: 660 mL/day for men and 330 mL/day for women. Men consumed 330 mL with lunch and 330 mL with dinner, whereas women consumed 330 mL with dinner. The same sex-specific total beverage volume was provided in all intervention groups: (i) the T-Beer group consumed regular lager beer containing 5.4% alcohol by volume (Alhambra Especial®, Granada, Spain); (ii) the T-Zero group consumed alcohol-free beer containing 0.0% alcohol (Cruzcampo®, Seville, Spain); (iii) the T-Ethanol group consumed sparkling water containing an amount of ethanol equivalent to that provided in the regular beer; and (iv) the T-Water group consumed sparkling water without added ethanol (Eliqua 2®, Font Salem, Spain).

At the beginning of each week, the research team provided each participant with the exact quantity of coded beverages assigned for that week and collected the empty bottles from the preceding week. Adherence was further monitored through weekly individual contact with the research staff. During these contacts, participants were asked to confirm consumption of the assigned beverage, absence of any additional alcohol intake, and compliance with the corresponding weekend instructions. Participants assigned to the alcohol-free groups were specifically required to confirm complete abstinence from alcohol throughout the intervention, whereas participants assigned to the alcohol-containing groups were instructed to consume only the prespecified amount and type of alcohol. Any missed intake or deviation from the assigned condition was to be documented. All participants included in the analyses reported full compliance, and no protocol deviations regarding beverage consumption were identified. Because there was no variability in reported adherence within the analytical sample, beverage compliance was not included as a covariate in the statistical models.

Usual alcohol consumption was assessed using the Beverage Intake Questionnaire. Dietary habits and habitual physical activity were also assessed using the MEDAS and IPAQ questionnaires, respectively (29–32). Participants assigned to the non-training group were instructed to maintain their usual dietary and physical activity habits during the intervention.

### 2.3 Experimental measurements

#### 2.3.1 HRV

Participants came to our laboratory in a motorized vehicle and avoided any physical activity since they woke up following specific study pre-conditions: (i) fasting conditions for more than 3 h, (ii) to abstain from alcohol intake and drugs or stimulant consumption 24 h before, and (iii) to avoid moderate-intensity (24 h) and vigorous-intensity physical activity (48 h) before the test. The environmental conditions were standardized, with a room temperature of 22–23 °C and relative humidity between 35% and 45%.

The R–R signal was recorded with the participant resting in the supine position on a standard padded examination couch. R-R signal recording lasted 15 min (after 10 min of acclimation before the assessment). To record the R-R signal, we used the Polar RS800CX (Polar Electro, Kempele, Finland). Participants were instructed not to talk or to move, and to relax as much as possible but to be awake. To download the R-R recordings, we used the Polar Pro Trainer 5 ® software (Polar Electro, Finland). R-R recordings were analyzed with the Kubios HRV Software, version 2.2 (University of Eastern Finland, Kuopio, Finland) (35), applying the medium filter provided by the Kubios HRV software and following the methodology described in previous studies (36, 37). We selected a 5 min interval, setting the smoothness prior approach with a Lambda value of 500, and a cubic interpolation at the default rate of 4 Hz, was used to remove not valid low-frequency baseline trend components. To obtain reproducible and valid data, HRV analyses were performed by the same trained researcher (38).

We obtained the time-domain HRV parameters SDNN, defined as the standard deviation of normal-to-normal R–R intervals, and RMSSD, defined as the root mean square of successive differences between normal-to-normal R–R intervals, as well as the Poincaré plot-derived parameters SD1 and SD2, using Kubios HRV software (1). SDNN is an index of global autonomic modulation (39), although resting short-term SDNN is related to vagal-activity (39). RMSSD is a measure of parasympathetic activity (39). From the Poincare plot, we obtained SD1, defined as the standard deviation of the points perpendicular to the line of identity, and SD2, defined as the standard deviation of Poincare plot along the line-of-identity. SD1 primarily reflects vagal modulation, whereas SD2 has been proposed as an inverse indicator of sympathetic activity, although its physiological interpretation remains debated (2, 40). The Stress score was computed as 1000*1/SD2; and the sympathetic/parasympathetic ratio was calculated as stress score/SD1 (2, 40). These indices have been proposed as indirect markers of sympathetic activity and autonomic balance, respectively (2, 40). Additional exploratory HRV parameters are presented as untransformed values in Supplementary Figure S1. The time-domain parameters included pNN50, defined as the percentage of successive normal-to-normal intervals differing by more than 50 ms and commonly considered an index of vagal modulation. The frequency-domain parameters included high-frequency power, defined within the 0.15–0.40 Hz range; low-frequency power, defined within the 0.04–0.15 Hz range; and the low-frequency/high-frequency ratio. High-frequency power primarily reflects vagal modulation, whereas low-frequency power reflects mixed autonomic and baroreflex influences. The low-frequency/high-frequency ratio was also calculated, although its interpretation as a measure of sympathovagal balance remains controversial (39).

To remove the HRV dependence on heart rate, we calculated corrected HRV parameters (36, 41) (see Supplementary Material, Figure S2), based on three assumptions: (i) if HRV parameters were negatively correlated with heart rate, the correction procedure consisted in calculating ratios between HRV parameters and different powers of the mean R-R interval; (ii) if HRV parameters were positively correlated with heart rate, the correction procedure was performed by multiplying HRV parameters by the adequate powers of mean R-R interval; and (iii) pre and post values of the different HRV parameters were normalized with the same powers of mean R-R intervals to calculate post-pre differences. We performed the next calculations: Corrected SDNN = SDNN/MeanRR^1.2^, Corrected RMSSD = RMSSD/MeanRR^1.9^, Corrected Stress Score = Stress Score*MeanRR^1^, Corrected Sympathetic/Parasympathetic Ratio = Sympathetic/Parasympathetic Ratio*MeanRR^2.9^.

### 2.4 Anthropometric and body composition measures

The weight and height were measured using an electronic scale (model 799, Electronic Column Scale, Hamburg, Germany). Lean mass and fat mass were evaluated by dual-energy x-ray absorptiometry (Discovery Wi, Hologic, Inc., Bedford, MA, USA) following the manufacturer's recommendations. We also calculated three indices of height-normalized body composition: Body Mass Index (BMI) as body weight/height^2^; lean mass index (LMI), as lean mass/height^2^; and fat mass index (FMI), as fat mass/height^2^. BMI, LMI, and FMI were expressed in kg/m^2^.

### 2.5 Statistical analysis

We used the Shapiro–Wilk test, visual check of histograms, and Q-Q plots to verify the distribution of all variables. The descriptive parameters were reported as mean (standard deviation), except in non-normal variables (i.e., Stress Score and Sympathetic/Parasympathetic ratio) where median (interquartile range) were detailed. The present ancillary study used a per-protocol, outcome-specific complete-case analytical approach. Participants assigned to the four training groups were eligible for analysis if they completed the intervention and attended at least 80% of the prescribed training sessions; this attendance criterion did not apply to participants assigned to the non-training group. For each HRV outcome, analyses included participants with valid data at both baseline and post-intervention for that specific outcome. Participants who withdrew, participants in the training groups who did not meet the attendance criterion, or participants with missing or invalid data at either assessment were excluded from the corresponding analysis. Missing HRV data were not imputed. The sample size of the parent BEER-HIIT trial was determined *a priori* assuming an effect size of 0.25, an *α* level of 0.05, and a statistical power of 85% (29). To account for an anticipated attrition rate of 20%, at least 16 participants per intervention group were planned (29). No separate *a priori* sample size calculation was performed specifically for the HRV outcomes evaluated in the present ancillary analysis. Because no beverage-related protocol deviations were documented among the participants included in the analyses, no adherence variable was entered as a covariate in the statistical models.

Non-normal variables (i.e., Stress Score and Sympathetic/Parasympathetic) were normalized using Napierian logarithm transformation. Before the intervention program, we performed an analysis of variance (ANOVA) to study baseline differences in heart rate, HRV parameters (i.e., SDNN, RMSSD, Stress Score and Sympathetic/Parasympathetic Ratio) and body composition variables (i.e., BMI, LMI, FMI) between intervention groups.

HRV was evaluated as a secondary outcome of the parent BEER-HIIT trial. No individual HRV parameter was designated as a primary outcome, and the trial registry did not specify a hierarchy among the different HRV parameters. For the present ancillary report, SDNN, RMSSD, stress score, and the sympathetic/parasympathetic ratio were treated as the main HRV outcomes. Analysis of covariance (ANCOVA) models adjusted for the corresponding baseline value were used to evaluate post-intervention between-group differences in each main HRV outcome, with Bonferroni adjustment applied to *post hoc* pairwise comparisons. Additional HRV parameters, including pNN50, high-frequency power, low-frequency power, and the low-frequency/high-frequency ratio, were considered exploratory and are presented in Supplementary Figure S1. Analyses using untransformed values and heart-rate-corrected values for the main HRV outcomes were considered supplementary sensitivity analyses and are presented in Supplementary Figures S1, S2, respectively. The F statistic, corresponding *p* value, and partial eta-squared effect size (*η*p^2^) were reported. All group-related changes were additionally adjusted by sex and age. *P* values of less than 0.05 were accepted to indicate statistical significance. All analyses were performed using the Statistical Package for Social Sciences (SPSS, v. 24.0, IBM SPSS Statistics, IBM Corporation). The graphical presentations were prepared using GraphPad Prism 7 (GraphPad Software, San Diego, CA, USA).

## 3 Results

**Figure 1:**
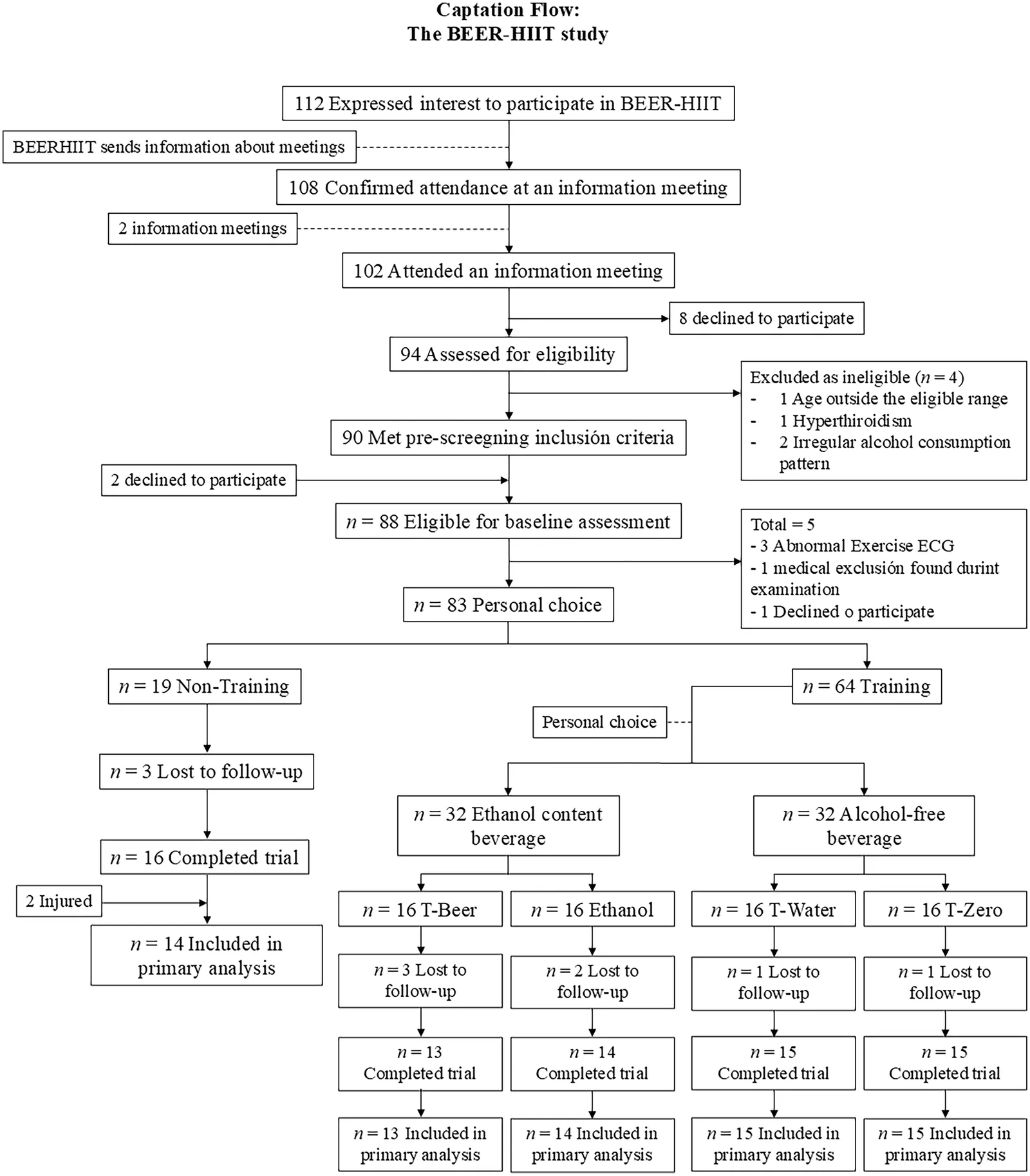
Participant flow through the BEER-HIIT study.

Figure 1 shows the flowchart of the BEER-HIIT study. Of the 83 participants assigned to a study group, 10 were lost to follow-up and two additional participants in the non-training group were excluded because of an injury. Therefore, 71 participants were included in the final analysis: 14 in N-T, 13 in T-Beer, 15 in T-Zero, 14 in T-Ethanol, and 15 in T-Water. The number of participants included in each outcome-specific analysis depended on the availability of valid baseline and post-intervention data.

**Figure 2:**
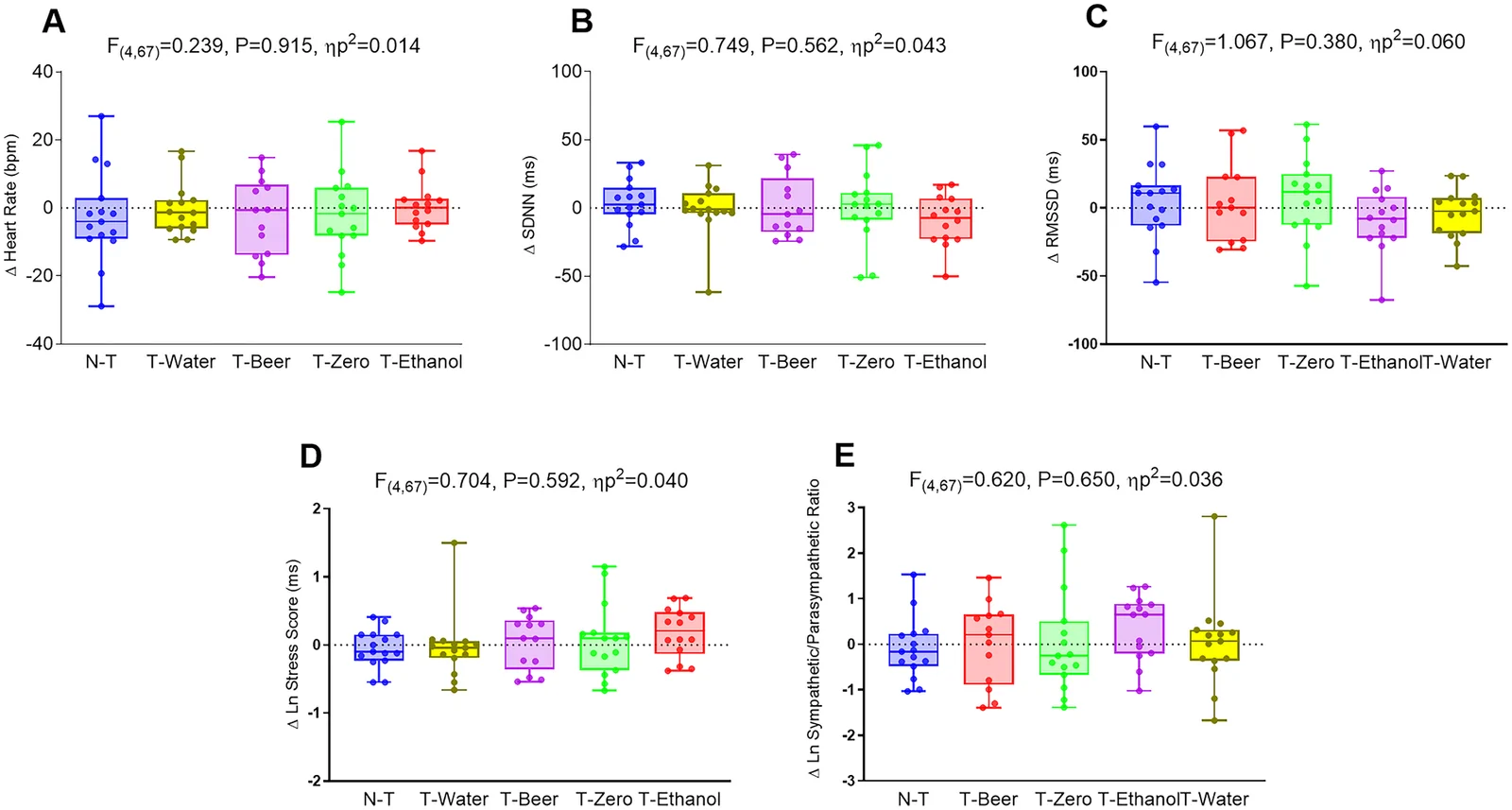
Changes in (**A**) heart rate, (**B**) SDNN, (**C**) RMSSD, (**D**) ln Stress Score, and (**E**) ln sympathetic-parasympathetic ratio after the intervention in the five groups.

The baseline characteristics of all participants are described in Table 1. No differences were observed in the baseline values among groups (all *p* > 0.079). Figure 2 shows changes in HRV parameters after the intervention study in the five groups. No significant differences of changes in heart rate (F = 0.239, *p* = 0.915, *η*p^2^ = 0.014), SDNN (F = 0.749, *p* = 0.562, *η*p^2^ = 0.043), RMSSD (F = 1.067, *p* = 0.380, *η*p^2^ = 0.060), Napierian logarithm of Stress Score (F = 0.704, *p* = 0.592, *η*p^2^ = 0.040) and Napierian logarithm of Sympathetic/Parasympathetic Ratio (F = 0.620, *p* = 0.650, *η*p^2^ = 0.036) were found after the intervention program. The results persisted after including sex and age in the model. Raw changes (i.e., not normalized using Napierian logarithm) in heart rate and HRV parameters after the intervention study in the five groups are shown in (Supplementary Material Figure S1; F ranges between 0.239 and 1.905; p ranges between 0.120 and 0.915; and *η*p^2^ ranges between 0.014 and 0.105). Also, in the Supplementary Material (Figure S2), are presented the changes in corrected HRV parameters, with no significant changes in HRV parameters in the different groups (F ranges between 0.588 and 2.383; p ranges between 0.061 and 0.673; and *η*p^2^ ranges between 0.034 and 0.130).

**Table 1:** Descriptive values segmented by intervention group.

Variables	All (*n* = 71)	Non training (*n* = 14)	T-Beer (*n* = 13)	T-Zero (*n* = 15)	T-Ethanol (*n* = 14)	T-Water (*n* = 15)	*p* Value
Age (years)	24.5 (6.2)	20.7 (3.0)	25.9 (6.1)	25.0 (6.5)	26.7 (7.0)	24.7 (6.6)	0.079
Sex (%)							
Men	34 (47.9)	7 (50.0)	6 (46.1)	6 (40)	7 (50)	8 (53.3)	
Women	37 (52.1)	7 (50.0)	7 (53.9)	9 (60)	7 (50)	7 (46.7)	
*Anthropometry and Body Composition*							
Weight (Kg)	68.6 (13.4)	62.7 (9.2)	70.0 (15.3)	71.5 (16.8)	69.7 (13.9)	69.4 (10.5)	0.431
Height (cm)	168.8 (8.4)	169.9 (9.6)	170.5 (5.6)	168.3 (10.7)	167.6 (8.1)	167.9 (7.4)	0.861
Body Mass Index (Kg/m^2^)	24.0 (3.7)	21.7 (2.0)	23.9 (4.2)	25.0 (3.7)	24.7 (4.2)	24.6 (3.3)	0.080
Lean Mass (Kg)	44.6 (9.7)	43.4 (7.7)	45.4 (11.3)	45.4 (11.9)	45.3 (10.4)	43.9 (8.0)	0.970
Lean Mass Index (Kg/m^2^)	15.2 (2.0)	14.9 (1.6)	15.5 (3.1)	15.8 (2.6)	15.97 (2.5)	15.5 (2.0)	0.802
Fat Mass Percentage (%)	30.0 (7.7)	26.1 (8.1)	29.8 (8.1)	32.2 (5.4)	30.1 (8.3)	31.9 (7.8)	0.198
Fat Mass Index (Kg/m^2^)	7.2 (3.3)	5.6 (2.1)	7.0 (2.5)	7.9 (1.8)	7.4 (2.8)	7.8 (2.6)	0.085
*HR and HRV parameters*							
Heart Rate (bpm)	69.0 (10.6)	69.7 (11.5)	69.1 (10.2)	68.5 (10.4)	67.9 (10.46)	69.7 (11.6)	0.990
SDNN (ms)	52.0 (22.9)	57.9 (24.4)	53.9 (16.6)	48.2 (18.9)	53.2 (25.81)	47.0 (27.9)	0.707
RMSSD (ms)	54.7 (29.8)	60.8 (30.9)	53.6 (19.9)	51.9 (24.9)	56.8 (38.12)	50.4 (34.0)	0.892
Stress Score (ms)	17.5 (10.9)	15.1 (11.1)	17.0 (7.0)	17.8 (10.1)	17.1 (16.5)	22.6 (24.1)	0.169
Sympathetic/Parasympathetic Ratio	0.49 (0.79)	0.31 (0.78)	0.55 (0.45)	0.48 (0.91)	0.52 (1.18)	0.84 (1.81)	0.125

T-Beer, HIIT plus regular beer; T-Zero, HIIT plus alcohol-free beer; T-Ethanol, HIIT plus ethanol in sparkling water; T-Water, HIIT plus sparkling water; HR, heart rate; HRV, heart rate variability; SDNN, standard deviation of normal-to-normal R–R intervals; RMSSD, root mean square of successive differences between normal-to-normal R–R intervals; HIIT, high-intensity interval training.

## 4 Discussion

This ancillary analysis of the BEER-HIIT study found no statistically significant between-group differences in HRV outcomes following a 10-week circuit-based HIIT program in healthy young adults. Similarly, no statistically significant differences in HRV responses were detected among the assigned beverage intervention groups. Importantly, the absence of detected adverse changes in HRV should not be interpreted as evidence that moderate alcohol consumption is safe or without broader health risks (20, 42, 43), because the present study was neither designed nor powered to evaluate the overall health consequences of alcohol consumption.

### 4.1 Beverage intervention and cardiac autonomic regulation

Previous observational studies have reported heterogeneous associations between alcohol consumption and cardiac autonomic regulation. Chronic heavy alcohol consumption has been associated with lower resting vagally mediated HRV parameters (26), whereas higher high-frequency power has been reported in a cross-sectional study of young habitual drinkers (28). These findings are not directly comparable with those of the present trial because of differences in study design, participant characteristics, alcohol dose, drinking pattern, duration of exposure, and control of beverage consumption. In the present study, healthy young adults consumed fixed, protocol-defined quantities of their assigned beverages over 10 weeks, while four of the five intervention groups also completed the HIIT programme.

Alcohol consumption may impair exercise adaptations through several mechanisms not captured by resting HRV. Excessive alcohol exposure can impair skeletal-muscle protein synthesis and anabolic signalling (44), and high-dose post-exercise alcohol ingestion has been shown to suppress myofibrillar protein synthesis even when protein is co-ingested (19). Alcohol may also alter sleep architecture and disrupt later-night sleep continuity (45) while high alcohol ingestion after exercise has also been associated with increased cortisol concentrations and a reduced testosterone-to-cortisol ratio during recovery (46). Nevertheless, these effects depend on the alcohol dose, timing, drinking pattern, and outcome assessed (21). Moreover, most previous experimental studies administered acute alcohol doses substantially higher than those prescribed in the present trial. Previous BEER-HIIT analyses did not identify an attenuation of physical fitness or body composition improvements under the present experimental conditions (18, 25). However, those findings do not exclude potential adverse effects on sleep, skeletal-muscle metabolism, endocrine function, recovery, or other outcomes that were not assessed.

The term “moderate alcohol consumption” is used in the present study solely to describe the prespecified experimental exposure and the operational definition adopted when the protocol was developed. It should not be understood as indicating a safe, beneficial, or recommended level of alcohol consumption. Although alcohol-related risk varies according to the amount and pattern of intake and the health outcome considered, no level of alcohol exposure can be regarded as completely risk-free, particularly with respect to carcinogenic effects (47). Alcohol consumption has also been associated with increased risks of several cancers (42, 43) and other adverse health outcomes, including cardiovascular and metabolic complications, impaired sleep, and compromised recovery following exercise (19–23, 42, 43, 48, 49). The present ancillary analysis evaluated selected cardiac autonomic outcomes over a 10-week period and was neither designed nor powered to determine the overall or long-term health consequences of alcohol consumption.

### 4.2 HIIT intervention and HRV

No statistically significant between-group differences in resting HRV were detected following the circuit-based HIIT programme. These results can be attributed to the specific methodological characteristics of the intervention, which are best contextualized within the FITT (frequency, intensity, time and type) variables of the training program. Regarding training frequency and time, the intervention comprised two weekly sessions over 10 weeks and provided approximately 20–33 min of exercise per session and 40–65 min of exercise per week. By comparison, previous studies reporting autonomic adaptations following high-intensity interval exercise have generally implemented approximately three weekly sessions over 12 weeks (50, 51). A systematic review of exercise interventions in healthy young and middle-aged adults similarly suggested that higher exercise intensities and frequencies may be more likely to improve HRV (52). However, training frequency was not evaluated quantitatively in that review, and substantial heterogeneity existed across exercise modalities, intervention characteristics, and HRV assessment methods.

Exercise session duration may represent an additional source of heterogeneity. Previous studies have employed sessions lasting approximately 30 min as well as longer sessions; however, session duration has varied alongside other training characteristics, making its independent contribution to chronic adaptations in resting HRV difficult to determine (52, 53). Evidence from acute-exercise studies suggests that longer exercise session duration may reduce HRV during exercise when accompanied by cardiovascular drift, whereas findings regarding its influence on post-exercise HRV recovery remain limited and conflicting (54). Therefore, whether sessions lasting approximately 30 min versus more than 30 min produce different chronic adaptations in resting HRV remains unclear.

Exercise intensity was prescribed using an RPE of 8–10 on a 10-point scale. However, the internal cardiovascular load was not continuously quantified using heart rate or oxygen consumption. Therefore, although the sessions were intended to provide a high-intensity stimulus, the cardiovascular demand experienced by each participant throughout the programme cannot be determined precisely. It is possible that the combined training frequency, volume, and internal load were insufficient to elicit detectable changes in resting HRV. This interpretation remains speculative, however, because these training components were not independently manipulated.

Exercise type may also have influenced the autonomic response. Endurance-oriented exercise training has frequently been associated with improvements in resting HRV parameters (15, 50–52), whereas the effects of resistance training on resting HRV have been less consistent (55, 56). The BEER-HIIT intervention consisted of eight weight-bearing exercises performed in a circuit format. Its cardiovascular and autonomic stimulus may therefore have differed from that produced by running- or cycling-based HIIT protocols involving a more sustained cardiovascular demand. Furthermore, the participants were healthy young adults with presumably preserved autonomic function, which may have limited the magnitude of measurable improvement. Nevertheless, because the individual components of the exercise prescription (i.e., FITT variables) were not experimentally compared and HRV was a secondary outcome, their contribution to the present findings cannot be established.

### 4.3 Clinical and practical interpretation

The present results do not provide evidence that this specific 10-week, twice-weekly, circuit-based HIIT programme improves resting HRV in healthy young adults. However, they should not be interpreted as demonstrating that circuit-based HIIT or other forms of high-intensity interval training cannot induce autonomic adaptations. Improvements in time-domain HRV parameters have been reported following a different whole-body, circuit-based HIIT protocol in insufficiently active young adults (17), indicating that autonomic responses may depend on the characteristics of the participant population and the specific exercise stimulus applied.

Accordingly, clinicians and exercise professionals should not prescribe the present protocol specifically for the purpose of improving resting HRV on the basis of these results alone. Nevertheless, the absence of detectable HRV adaptations does not preclude benefits in other outcomes. Improvements in physical fitness and body composition have previously been reported in the BEER-HIIT trial (18, 25), emphasizing that adaptations across different physiological systems do not necessarily occur in parallel.

### 4.4 Limitations

Our study has several limitations. First, HRV was a secondary outcome, no specific *a priori* sample-size calculation was performed, and the relatively small group sizes may have limited the ability to detect small or moderate effects. Thus, type II error cannot be excluded, and null findings should not be interpreted as evidence of no effect. Second, the sample comprised of healthy adults aged 18–40 years with presumably preserved autonomic function, limiting generalizability to older adults and populations with impaired autonomic regulation. Third, the Polar RS800CX is not the gold standard for HRV assessment, although validity and reliability have been reported (57, 58). Respiratory frequency was not controlled during the HRV recordings; however, its influence on vagally mediated resting HRV appears to be limited under standardized resting conditions (36). In addition, the internal cardiovascular load of the HIIT sessions was not continuously quantified using heart rate or oxygen consumption. Fourth, dietary habits, physical activity, and usual alcohol intake were assessed by questionnaire, without continuous or objective monitoring. Participants did not complete daily alcohol-consumption diaries, and beverage adherence was not biochemically verified. Although controlled weekly beverage provision, empty-bottle collection, and regular contact with the research team supported adherence, unreported deviations from the protocol cannot be excluded. Fifth, participants initially selected whether they were willing to receive an alcohol-containing or alcohol-free beverage and were subsequently randomized within their selected beverage category. This procedure avoided assigning alcohol consumption contrary to participants preferences but precluded complete randomization across all intervention groups and may have introduced selection bias. Double blinding was also not feasible because of differences in the organoleptic properties of the beverages. Finally, the per-protocol complete-case analysis excluded participants with missing or invalid HRV data, did not impute missing values, and may therefore have introduced attrition or selection bias.

## 5 Conclusion

In this ancillary analysis of the BEER-HIIT study, no statistically significant between-group differences in HRV outcomes were detected following a 10-week circuit-based HIIT program involving weight-bearing exercises in healthy young adults. Similarly, no statistically significant differences in HRV responses were observed among the assigned beer, alcohol-free beer, ethanol, and water intervention groups during the 10-week HIIT program. Given that HRV was a secondary outcome and the study may have been insufficiently powered to detect small or moderate effects, these findings should not be interpreted as evidence of the absence of an effect. Furthermore, the lack of statistically significant adverse differences in HRV among the beverage intervention groups should not be interpreted as evidence that alcohol consumption is safe or without broader health risks, and alcohol intake should not be recommended on the basis of these findings. Future adequately powered studies are needed to investigate HRV responses to exercise and alcohol-containing beverage interventions, particularly in older adults and populations with impaired autonomic regulation or pre-existing health conditions.

## Data Availability

The raw data supporting the conclusions of this article will be made available by the authors, without undue reservation.

## Supplementary material

Supplementary Figure S1. Raw changes in heart rate (Panel A) and heart rate variability parameters (Panel B, C, D, E, F, G, H and I) after the intervention study in the five groups.

Supplementary Figure S2. Raw changes in corrected heart rate variability parameters (Panels A, B, C and D) after the intervention study in the five groups.
